# Detection of chronic lymphocytic leukemia subpopulations in peripheral blood by phage ligands of tumor immunoglobulin B cell receptors

**DOI:** 10.1038/s41375-020-0885-y

**Published:** 2020-06-01

**Authors:** Selena Mimmi, Domenico Maisano, Nancy Nisticò, Eleonora Vecchio, Federico Chiurazzi, Katia Ferrara, Marialuigia Iannalfo, Alessandro D’Ambrosio, Giuseppe Fiume, Enrico Iaccino, Ileana Quinto

**Affiliations:** 1 Department of Experimental and Clinical Medicine, “Magna Græcia” University of Catanzaro, Campus “S. Venuta”, Viale Europa, Germaneto, 88100 Catanzaro, Italy; 2 Department of Clinical Medicine, University “Federico II” of Naples, Naples, Italy

**Keywords:** Chronic lymphocytic leukaemia, Lymphoproliferative disorders

## To the Editor:

The B cell receptor (BCR) is an immunoglobulin (Ig) expressed on the membrane surface of mature B cells [1]. The IgBCR has two heavy and two light chains, each one made by a constant and a variable region. The variable sequences of the IgBCR are generated during B-cell differentiation through somatic recombination, so called VDJ recombination, and somatic hypermutation [2]. Once assembled on the B-cell surface, the IgBCR recognizes a specific antigen through its binding to the variable regions. This event triggers the B-cell immune response against the antigen. Since the variable regions are B cell specific, the sequence of IgBCR allows the identification of single B-cell clones [3]. In B-lymphoproliferative disorders, the IgBCR plays a key role in the development, proliferation, and survival of tumor B cells [4], by an antigen-driven process that triggers a molecular cascade of events that lead to transcriptional activation of proliferative and antiapoptotic genes [5–7].

Chronic lymphocytic leukemia (CLL) is a B-proliferative disorder characterized by a clonal expansion and accumulation of neoplastic CD19/CD5/CD23/CD20-positive B-lymphocytes in blood, bone marrow, and other tissues [8]. Several studies support the hypothesis that a common pool of environmental antigens or self-antigens drives the selection of tumor B cells through the persistent triggering of IgBCR [9, 10]. Consistently, the sequence analysis of VDJ rearrangement of tumor IgBCRs revealed a high level of homology in more than 30% of CLL patients, defined as stereotyped IgBCR, with the prevalence of VH1, VH3, and VH4 families [11, 12]. CLL are defined as mutated (M-CLL) or unmutated (U-CLL) depending on the mutational rate of the IgBCR variable regions, being more or less than 2% respect to the germline, respectively [11, 12]. U-CLL cells expressing the VH1-69 rearrangement usually have an inducible IgBCR and show an aggressive behavior [11, 12]. In this regard, it would be useful to develop new molecular tools for rapid detection of aggressive CLL clones in peripheral blood.

We previously used phage display for identifying peptide ligands of B-lymphoma, multiple myeloma, and CLL IgBCRs [3, 13–15]. In CLL patients, we documented the co-existence of different CLL clones in a single patient as detected by phage-expressed peptide ligands of the tumor IgBCRs [3, 15]. In this study, we used the phage display for identifying a peptide sequence that was commonly recognized by VH1-69 U-CLL clones of two CLL patients. These patients, named CLL#1 and CLL#5, were randomly referred to the Hematology Unit—University Federico II of Naples and initially diagnosed as CLL Binet stage A. At month 8, CLL#1 worsen to Binet stage C and returned to Binet stage A after 6 months of therapy. CLL#5 was stably CLL Binet stage A for 2 years of observation. In the course of disease, we analysed four blood samples of patient CLL#1 (CLL#1 a–d) and three blood samples of patient CLL#5 (CLL#5 a–c). Clinical and laboratory data are reported in Supplementary Table S1. Total RNA was extracted from purified B cells and reverse transcribed in cDNA followed by nested RT-PCR to amplify the heavy and light chains of the Ig variable regions, as previously described [3] (Supplementary Fig. S1). The PCR-amplified products were appropriately digested and cloned in expression vectors for DNA sequencing. The Ig nucleotide sequences were analysed by the international ImMunoGeneTics information system® (IMGT® http://www.imgt.org) in order to find a match with the relative VH and VL families, according to the classification of the stereotyped IgBCRs. In both CLL patients, we observed the co-existence of variable VH nucleotide sequences, both U-CLL and M-CLL, with a unique VL nucleotide sequence at each time of collection (Table 1) (GenBank accession numbers MT334403 to MT334414).

**Table 1:** VH and VL families of CLL IgBCRs.

		Heavy chain					Light chain	
Patient	Sample collection time	V_H_DJ_H_	VH Mutational status	VH CDR3 aa number	VH CDR3 aa sequence	(%)	V_L_J_L_	(%)
CLL#1	CLL#1a (month 1)	V1-69/D3-16*01/J6*02	U-CLL	20	CARDLGMITFGGDYYYYGMDVW	60	V3-1*01/J3*02	100%
		V4-4/D2-21*01/J6*03	U-CLL	18	CARVVVIVTIRGYNYYMDVW	40		
	CLL#1b (month 5)	V1-69/D3-16*01/J6*02	U-CLL	20	CARDLGMITFGGDYYYYGMDVW	50		
		V3-21*01/D3-10*01/J3*02	M-CLL	14	CARDYGSGRSPPQNIW	20		
		V3-15*07/D2-21*01/J3*02	M-CLL	16	CTTAPKESRLPWEAFDIW	10		
		V3-30*03/D5-12*01/J3*02	M-CLL	20	CARGQEVDTVSKILYADTLDIW	10		
		V4-59*01/D3-22*01/J3*02	U-CLL	17	CARGLLYYDSSGYQAFDIW	10		
	CLL#1c (month 8)	V1-69/D3-16*01/J6*02	U-CLL	20	CARDLGMITFGGDYYYYGMDVW	80		
		V4-59*08/D6-13*01/J4*02	U-CLL	18	CARDRWYSSSYYGGYYFDYW	10		
		V5-10*03/D6-19*01/J4*02	U-CLL	12	CARHRHSSGFGDYW	10		
	CLL#1d (month 24)	V3-53*02/D3-09*01/J4*02	M-CLL	11	CVSGYDSAKLASW	70		
		V1-69/D3-16*01/J6*02	U-CLL	20	CARDLGMITFGGDYYYYGMDVW	30		
CLL#5	CLL#5a (month 1)	V1-69/D7-27*01/J3*02	U-CLL	12	CARSAYWGYFDIW	75	V1-33*01/J3*01	100%
		V3-7*03/D1-7*01/J4*02	M-CLL	9	CARDNWNYVYW	25		
	CLL#5b (month 12)	V1-69/D7-27*01/J3*02	U-CLL	12	CARSAYWGYFDIW	45		
		V3-49/D3-22/J3*02	U-CLL	20	CTRGPPYDSSGNYLRLDAFDIW	30		
		V4-4*02/D2-21/J6*03	U-CLL	18	CARVVVIVTIRGYNYYMDVW	30		
		V3-7*03/D1-7*01/J4*02	M-CLL	9	CARDNWNYVYW	5		
	CLL#5c (month 24)	V1-69/D7-27*01/J3*02	U-CLL	12	CARSAYWGYFDIW	35		
		V3-49/D3-22/J3*02	U-CLL	20	CTRGPPYDSSGNYLRLDAFDIW	25		
		V4-4*02/D2-21/J6*03	U-CLL	18	CARVVVIVTIRGYNYYMDVW	25		
		V3-7*03/D1-7*01/J4*02	M-CLL	9	CARDNWNYVYW	15		

Blood samples were collected from patients CLL#1 and CLL#5 at the indicated time. Total RNA was extracted from purified B cells, and the IgBCR variable regions were amplified by RT-PCR, cloned in expression vectors AbVec-hIgG1 (GenBank: FJ475055.1) for the heavy chain and AbVec-hIgLambda (GenBank: FJ517647.1) for the light chain, and subject to DNA sequencing. Genetic rearrangements of the heavy (VH) and light (VL) chains were analyzed according to the International ImMunoGeneTics information system® (http://www.imgt.org). Status is defined unmutated (U-CLL) or mutated (M-CLL) based on mutational differences (<2% or >2%, respectively) compared with the germline sequence. Percentage indicates the frequency of the clone expressing the indicated VDJ rearrangement of heavy chain.

Among the total CLL populations, an unmutated VH1-69 CLL subpopulation persisted in the patient CLL#1 (VH1-69/D3-16*01/J6*02) and patient CLL#5 (VH1-69/D7-27*01/J3*02) all time of observation, ranging between 30 and 80% of total CLL population. Other CLL clones were either mutated or unmutated, appearing and disappearing over time at a lower percentage (Table 1). In the patient CLL#1, the VH1-69 clone peaked at 80% at passage to Binet stage C and dropped to 30% after therapy with the remission of disease (Table 1). This evidence indicated that the VH1-69 U-CLL clone had an aggressive behavior as, differently from the other CLL clones, it persisted elevated during the time of observation.

**Fig. 1:**
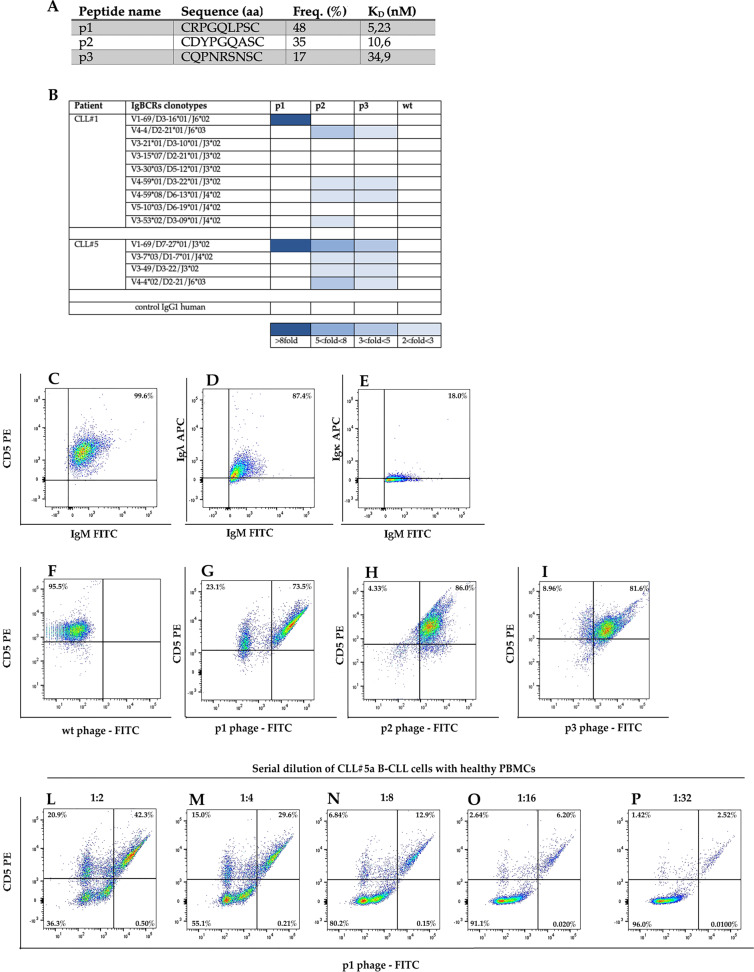
Analysis of phage ligands of CLL IgBCR in patients CLL#1 and CLL#5. **a** Phages ligands of VH1-69 U-CLL IgBCR of CLL#5 patient. RPL screening was performed using the recombinant VH1-69 U-CLL Ig of patient CLL#5 as bait. The phages p1, p2, and p3 were selected at the indicated frequency; DNA sequencing determined the amino acid (aa) sequence of the insert random peptide. The affinity binding was measured as K_D_ by Scatchard Plot analysis of the experiment shown in Supplementary Fig. S4. **b** Profile of phage binding to the CLL IgBCRs of patient CLL#1 and CLL#5. The recombinant IgBCR (10 ng/μl) was incubated in 96-microwells with the indicated phages (1 × 10^9^ PFU/μl). The phage binding was revealed by the anti-M13 HRP conjugated antibody (Abcam—UK) and relative enzyme substrate. Absorbance was calculated at 405 nm by the MultiskanTM GO Microplate Spectrophotometer (Thermo Fisher Scientific—USA). Wild-type phage and a human IgG were included as controls. Dark blue corresponds to the highest absorbance (>8-fold respect to the blank); decreasing shades of blue correspond to lower absorbance values, as indicated at the bottom of the table. White squares indicate lack of binding. **c**–**p** Phage-based flow cytometry of CLL clones. B cells of patient CLL#5 collected at month 1 were analyzed by flow cytometry for the expression of CD5, IgM, and the Igλ and Igκ light chains **c**–**e**. The same B-cell sample was analyzed for CD5 expression and phage binding by incubation with the phages wild type (wt) (**f**), p1 (**g**), p2 (**h**), and p3 (**i**). The B-CLL sample was serially diluted with healthy PBMCs (1:2, 1:4, 1:8, 1:16, 1:32) and analyzed for the binding of phage p1 (**l**–**p**). Data were acquired by FACS Canto II (Miltenyi Biotec—Germany) and analyzed by FlowJo Software.

We reasoned that a specific ligand could be a potential tool to discriminate and monitor the VH1-69 U-CLL subpopulation in peripheral blood. To this end, we produced the recombinant CLL IgBCRs, as previously described [3] (Supplementary Fig. S2). The purified VH1-69 U-CLL Ig of patient CLL#5 was used as bait to screen an M13 phage-displayed random peptide library (RPL) [3, 16]. The workflow of phage selection is shown in Supplementary Fig. S3. Twenty-five phage colonies were selected and subject to DNA sequencing to determine the amino acid sequence required for the binding to the VH1-69 U-CLL Ig. Three amino acid sequences were identified at different frequencies: p1 (48% phages), p2 (35% phages), and p3 (17% phages) (Fig. 1a). By ELISA, the phage p1 showed higher affinity binding to VH1-69 U-CLL Ig of patient CLL#5 compared with phages p2 and p3 (Fig. 1b) (Supplementary Fig. S4). Further, the phage p1 did not bind the other three CLL clones of patient CLL#5, while phage p2 and p3 did (Fig. 1b). We then analysed the binding of the three phages to the CLL Igs of patient CLL#1. The phage p1 bound only the VH1-69 U-CLL Ig of CLL#1 with high affinity (Fig. 1b). Differently, the phages p2 and p3 did not bind VH1-69 U-CLL Ig of CLL#1 and showed a weak binding to other Igs of patient CLL#1 (Fig. 1b). These results indicated that the phage p1 exclusively bound the VH1-69 U-CLL IgBCR of both patients CLL#1 and CLL#5 (Fig. 1b).

Being the VH1-69 rearrangement of IgBCR often associated with poor prognosis of CLL [12], we considered the possibility to detect the VH1-69 U-CLL population in peripheral blood using phage p1 as specific ligand. To this end, we analysed the immunophenotype and phage binding profile of B cells collected from patient CLL#5 at month 1. Consistently with clinical data (Supplementary Table S1), B cells were 99.5% CD5-positive and expressed the IgM isotype and the lambda light chain (Fig. 1c–e). As control, the phage wild type did not show any binding (Fig. 1f). The phage p1 detected 73.5% of CD5-positive cells, and the phage p2 and phage p3 detected 86.0% and 81.6%, respectively (Fig. 1g–i). These results were consistent with the occurrence of 75% VH1-69 U-CLL clones in the patient CLL#5 as detected by VDJ sequence (Table 1) and the specific binding of phage p1 to the relative VH1-69 U-CLL IgBCR (Fig. 1b). The progressive dilution of B-CLL cells with healthy PBMCs caused a parallel decrease of phage p1-positivity, confirming the specific binding of the phage p1 to the target tumor clones (Fig. 1l–p). Thus, the phage p1 revealed to be a specific probe for detecting the VH1-69 U-CLL population within a mix of blood cells.

CLL harbors different tumor clones in a single CLL patient, which can be identified by the IgBCR structure. Sequencing the tumor IgBCRs determines the variability of tumor clones but is not useful for flow cytometry of tumor clones in real time. To date, the diagnosis of disease is based on the evaluation of total number of CD5-positive cells associated with the stage of lymphoid tissues infiltration. In this context, it would be relevant to have sensitive molecular tools for monitoring the different tumor clones based on IgBCR recognition. This would be possible by developing new reagents for flow cytometry in order to detect and eventually isolate the most aggressive CLL subpopulations for molecular characterization. Our study opens to the possibility to monitor CLL clones in the peripheral blood of patients by using phage ligands as specific probes of CLL subpopulations. In perspective, the validation of this experimental approach on a large number of patients could provide a new method of clusterization of CLL clones based on their epitopic reactivity.

## Supplementary information

Supplementary Materials and Methods

Supplementary Table S1. Clinical and laboratory data of patients CLL#1 and CLL#5

Supplementary Figure S1. RT-PCR analysis of variable regions of CLL IgBCRs.

Supplementary Figure S2. Purification of recombinant VH1-69 U-CLL-IgG.

Supplementary Figure S3. Workflow of the RPL screening.

Supplementary Figure S4. Binding assay of phages 1, 2 and 3 to the VH1-69 U-CLL IgG of patient CLL#5.
