# Supplementary material for: Detection of chronic lymphocytic leukemia subpopulations in peripheral blood by phage ligands of tumor immunoglobulin B cell receptors
Source: Leukemia. 2020 Jun 1;35(2):610–4. doi: 10.1038/s41375-020-0885-y (PMC7862058; doi:10.1038/s41375-020-0885-y)
Supplement: Supplementary file 1 — Supplementary Materials and Methods [file 41375_2020_885_MOESM1_ESM.pdf]

Supplementary Materials and Methods

B-CLL cells isolation.

Peripheral blood samples were collected from two diagnosed CLL patients (CLL#1 and CLL#5), following written informed consent and approval by the local ethics committees of the University "Federico II" of Naples, Italy. Clinical and biological data of these from patients are summarized in Supplementary Table S1. Blood samples were centrifuged by density gradient centrifugation using Ficoll-Paque PLUS (Sigma Aldrich-Germany) for total cell collection, and B-cells were isolated by negative selection with the B-CLL Cell Isolation Kit, human (Miltenyi Biotec – Germany). Cell aliquots ($1x10^6$ cells) of each collection were kept in liquid nitrogen frozen.

Cloning and DNA sequencing of IgBCR variable regions.

Total RNA was extracted from B-cells ($1x10^6$ cells) using TRIzol™ Reagent (Thermo Fisher Scientfic – USA). RNA was reverse transcribed using the 5X iScript RT Superscript (BioRad Laboratoires, Italy) and the variable regions of heavy (IgH) and light (Igκ or Igλ) were amplified by nested PCR using primer mixes, as previously described[1]. PCR products were analyzed by agarose gel electrophoresis and purified using PCR Purification Kit (QIAGEN S.p.A. - Italy), according to the manufacturer's instructions. The PCR products were digested with appropriate restriction enzymes (AgeI and Sall for IgH; AgeI and BsiWI for Igκ; AgeI and XhoI for Igλ) and after purification with the Gel purification kit (QIAGEN S.p.A. - Italy) the digested products were ligated into the expression vectors γ1 HC, κ LC and λ LC (gift of Dr. Michel Nussenzweig-The Rockefeller University Hospital, NY, USA; GenBank accession n. DQ407610). The ligation products were transfected in DH5alpha competent bacterial cells (Thermo Fisher Scientific – USA, cat.n. 18258012) and transfected bacterial colonies were selected and singularly purified in ampicillin-resistance medium. DNA was extracted from 25 randomly single colonies and amplified by PCR using a primer mix, as described[1]. The PCR products related to the variable regions of IgH, Igκ or Igλ were sequenced and analyzed according to the International ImMunoGeneTics information system® (http://www.imgt.org), including gene usage, number of amino acids changes due to somatic hypermutation, and amino acids length of VH CDR3.

Production of monoclonal IgG.

Plasmid vectors (10µg each) containing the variable regions of IgH, and Igκ or Igλ, were co-transfected in human embryonic kidney (HEK) 293T cells (ATCC® CRL-3216™) by

calcium-phosphate precipitation. Transfected cells were cultured for 7 days in 10ml serum-free DMEM supplemented with 1% Nutridoma-SP (Roche - Germany). Culture medium containing the secreted IgGs was collected, centrifuged for 5 minutes at 400 x *g* at room temperature and filtered with a 0.2 micrometer sterile filter. Secreted IgGs were purified from culture medium by affinity chromatography using a G protein affinity column (GE Healthcare - Germany), according to manufacturer's instructions. Purified IgGs were visualized by SDS-PAGE with Coomassie blue staining. Briefly, protein bands were separated in a 10% polyacrylamide gel under reducing (with β-mercaptoethanol) and non-reducing (without β-mercaptoethanol) conditions. Gel was first fixed with a fixing solution (50% Methanol + 10% Acetic Acid in distillated water) and then stained over night at 4°C with Coomassie Brilliant Blue G-250 solution (0,006% Coomassie Brilliant Blue G-250 + 10% Acetic Acid in distillated water). Purified IgGs were quantified by ELISA. Briefly, 96-well microplate (Thermo Fisher Scientific – USA) was coated with different amounts of purified IgGs (from 10μl to 0.001μl) in carbonate buffer pH9.0 overnight at 4°C; a human IgG1κ antibody (Sigma Aldrich -Germany, cat.n. I5154) at increasing concentrations (10ng/μl up to 10ρg/μl) was included as standard. After 1-hr incubation at 37°C, unbound proteins were washed out with washing buffer (PBS 1X + 0.005% $Tween_{20}$). The remaining protein-binding sites in the coated wells were blocked by addition of 100μl blocking buffer (PBS 1X, 0.005% $Tween_{20}$, 5% BSA). IgGs were detected by adding an anti-Human IgG HRP-conjugated (Sigma Aldrich -Germany, cat.n. AP113P) and relative substrate. Absorbance was calculated at 405nm using the Multiskan™ GO Microplate Spectrophotometer (Thermo Fisher Scientific – USA).

Phage display screening

The selection of phage ligands of CLL Ig was performed by screening of a random peptide library expressed in the minor coat protein pIII of the filamentous phage M13 (Ph.D.TM-C7C Phage Display Peptide Library - New England Biolabs - NEB)[1,2]. Briefly, a 96-well microplate (Thermo Fisher Scientfic – USA) was coated with the purified IgGs (10ng/μl) in carbonate buffer pH9.0 overnight at 4°C. After washing out the unbound IgGs and blocking the remaining protein-binding sites with blocking buffer, the recombinant phages ($1x10^{10}$) were added for 1 hr–incubation at 37°C. Unbound phages were washed out, and the bound phages were eluted with 200 μL of elution buffer (0.2M glycine-HCl pH 2.2) and adjusted to pH 7 with 1M Tris pH 9.0. Eluted phages were measured by plaque forming unit (PFU/ml), and amplified in K12 *E.coli* cells for additional 3 rounds of affinity purification. Selected phages were purified by precipitation in polyethylene glycol (PEG MW.8000 - Sigma Aldrich - Germany) solution and measured by titration. 25

randomly single phage clones were isolated and purified, as previously described[1]. Phage single strand DNA was isolated by phenol-chloroform extraction followed by precipitation in ethanol solution, according to standard protocol. The nucleotide sequence corresponding to the inserted random peptide was amplified and identified using the - 96 gIII sequencing primer by standard Sanger sequencing method.

Enzime-linked immunosorbent assay.

Enzime-linked immunosorbent assay (ELISA) was used to measure the binding of selected phages to the IgGs. Briefly, a 96-well microplate (Thermo Fisher Scientfic – USA) was coated with the purified IgGs (10ng/µl) in carbonate buffer pH9.0 overnight at 4°C. After washing out the unbound IgGs and blocking the remaining protein-binding sites with blocking buffer (PBS 1X, 0.005% $Tween_{20}$, 5% BSA), different amounts of single purified phages (from $1x10^9$ PFU/µl to $1x10^{-3}$ PFU/µl) were added. Phage binding was revealed by the anti-M13 HRP conjugated antibody (Abcam – UK, cat.n. ab50370) and relative enzyme substrate. Absorbance was calculated at 405nm by the Multiskan™ GO Microplate Spectrophotometer (Thermo Fisher Scientific -USA). The K_D values were calculated by Scatchard Plot analysis using Prism software. The wild type M13 phage (without peptide insert) and a human immunoglobulin IgG1 (Sigma Aldrich -Germany, cat.n. I5154) were included in ELISA as negative controls. Absorbance values (mean ± SEMs) of 3 independent experiments are shown in Figure 1B and Supplementary Figure S4

Flow cytometry.

The immunophenotype of B cells was analyzed by labelling with anti-CD5 PE (Miltenyi Biotec – Germany, cat.n. 130-110-990), anti IgM-FITC (Thermo Fisher Scientific – USA cat. N. 11-9998-42), anti- Igλ- APC (Miltenyi Biotec – Germany, cat.n. 130-093-038), and anti-Igκ APC (Miltenyi Biotec – Germany, cat.n. 130-105-373). Briefly, B CLL cells (x 10^6) were washed with PBS 1X, 20 min-incubated with the indicated antibodies on ice, washed with PBS 1X, and suspended in FacsFlow Buffer (BD – Italy).

For phage binding analysis, B-CLL cells ($1x10^5$) undiluted or diluted (1:2, 1:4, 1:16, 1:32) with healthy PBMCs were incubated with phage ($1x10^6$) and anti-CD5 PE (Miltenyi Biotec – Germany, cat.n. 130-110-990) for 20 minutes on ice. After washing with PBS1X, cells were incubated with an anti-M13 biotin conjugated antibody (Abcam – UK, cat.n. ab17269) for 20 minutes on ice. The binding was detected using FITC-conjugated streptavidin (Thermo Fisher Scientific -USA, cat.n. 434311) in FacsFlow Buffer. For setting the gate of B-CLL cells, anti CD19-APC (Miltenyi Biotec – Germany, cat.n. 170-078-090) and anti-CD5-PE

(Miltenyi Biotec – Germany, cat.n. 130-110-990) antibodies were used. Data were acquired by FACS Canto II (Miltenyi Biotec – Germany) and analyzed by FlowJo Software.

References of Supplementary Materials and Methods

1) Mimmi, S., Vecchio, E., Iaccino, E. et al. Evidence of shared epitopic reactivity among independent B-cell clones in chronic lymphocytic leukemia patients. *Leukemia* 30, 2419–2422 (2016)

2) Mimmi S, Maisano D, Quinto I, Iaccino E. Phage Display: An Overview in Context to Drug Discovery. *Trends Pharmacol Sci*. 40(2):87-91 (2019)
