# Supplementary material for: Detection of chronic lymphocytic leukemia subpopulations in peripheral blood by phage ligands of tumor immunoglobulin B cell receptors
Source: Leukemia. 2020 Jun 1;35(2):610–4. doi: 10.1038/s41375-020-0885-y (PMC7862058; doi:10.1038/s41375-020-0885-y)
Supplement: Supplementary file 2 — Supplementary Table S1. Clinical and laboratory data of patients CLL#1 and CLL#5 [file 41375_2020_885_MOESM2_ESM.pdf]

Supplementary Table S1. Clinical and laboratory data of patients CLL#1 and CLL#5.

Patients CLL#1 and CLL#5 were clinically followed for 2 years, and peripheral blood samples were collected at the indicated time. At the diagnosis, CLL#1 patient counted 98% CD19/CD5 positive B cells, and CLL#5 patient counted 82% CD19/CD5 positive B cells. Definition of CLL stage was according to Binet classification (Cancer.Net Editorial Board, 10/2017). Cytogenetic analysis was performed using Fluorescent in situ hybridization (FISH) with probes XL p53/LSI ATM and XL DLEU/LAMP/12cen (MetaSystems). WBC, white blood cells.

Patient	Sample (collection time)	WBC	Binet stage	Cytogenetics	Therapy
CLL#1 65-years old male	CLL#1a (month 1)	40,410/mmc	A	Del13q14	None
	CLL#1b (month 5)	69,070/mmc	A		None
	CLL#1c (month 8)	92,670/mmc	C		rituximab-bendamustine (for 6 months)
	CLL#1d (month 24)	6,700/mmc	A		None
CLL#5 80-years old female	CLL#5a (month 1)	57,210/mm,	A	Del13q14	None
	CLL#5b (month 12)	119,000/mmc	A		None
	CLL#5c (month 24)	86,500/mmc	A		None
