# Supplementary material for: Detection of chronic lymphocytic leukemia subpopulations in peripheral blood by phage ligands of tumor immunoglobulin B cell receptors
Source: Leukemia. 2020 Jun 1;35(2):610–4. doi: 10.1038/s41375-020-0885-y (PMC7862058; doi:10.1038/s41375-020-0885-y)
Supplement: Supplementary file 3 — Supplementary Figure S1. RT-PCR analysis of variable regions of CLL IgBCRs. [file 41375_2020_885_MOESM3_ESM.pdf]

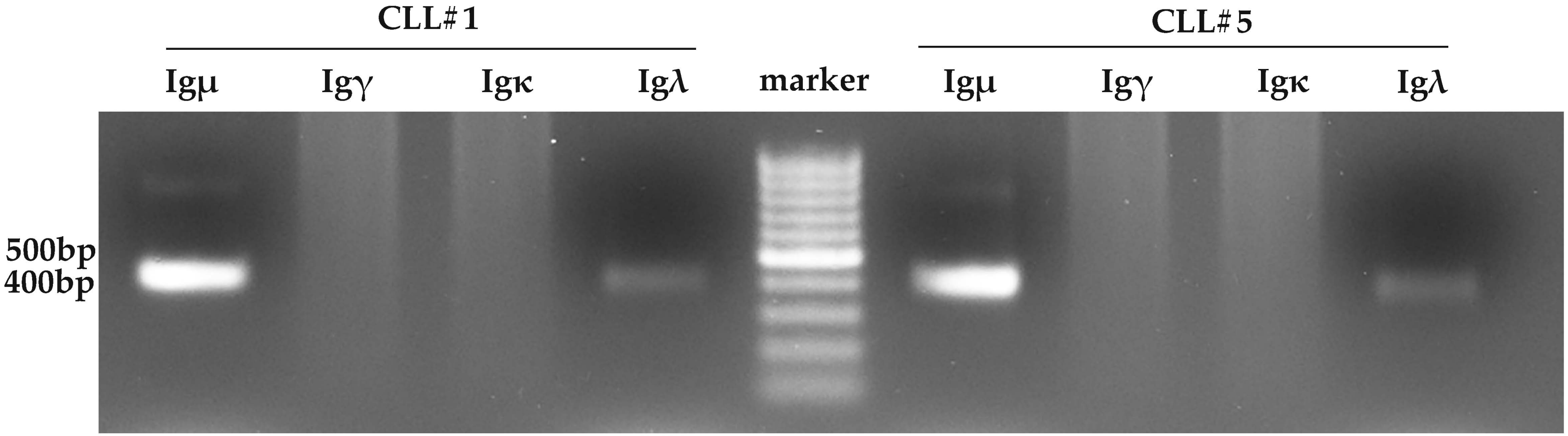

Supplementary Figure S1. RT-PCR analysis of variable regions of CLL IgBCRs.

Total RNA was extracted from B cells of patients CLL#1 and CLL#5. The variable genes of heavy and light chains of IgBCR were RT-PCR amplified with appropriate primers and analysed by electrophoresis on 1.5% agarose gel. In both patients, the Igμ (450 bp) and the Ig*λ* variable genes (405 bp) were detected; PCR products were not obtained for Ig**γ** or Ig*κ*. These results indicated that the CLL IgBCR populations of both patients expressed an immunoglobulin containing the Igμ and Ig*λ* chains. The Gene Ruler 100bp (Thermo Fisher Scientfic – USA) was included as marker.
