# Supplementary material for: Detection of chronic lymphocytic leukemia subpopulations in peripheral blood by phage ligands of tumor immunoglobulin B cell receptors
Source: Leukemia. 2020 Jun 1;35(2):610–4. doi: 10.1038/s41375-020-0885-y (PMC7862058; doi:10.1038/s41375-020-0885-y)
Supplement: Supplementary file 4 — Supplementary Figure S2. Purification of recombinant VH1-69 U-CLL-IgG. [file 41375_2020_885_MOESM4_ESM.pdf]

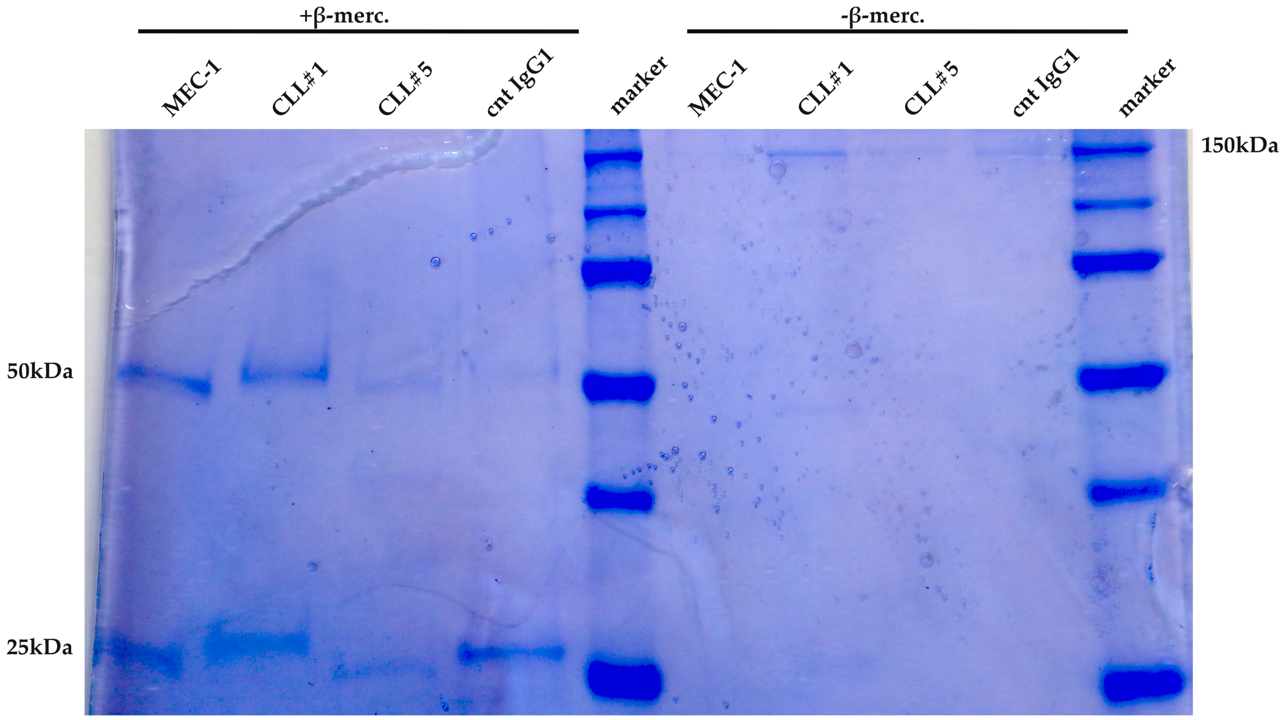

Supplementary Figure S2. Purification of recombinant VH1-69 U-CLL-IgG.

The heavy (50kDa) and light (25kDa) chains of the VH1-69 U-CLL IgBCR of patients CLL#1 and CLL#5 were co-transfected in HEK 293T cells. The secreted IgGs (150kDa) were purified from the cell culture by affinity chromatography and separated by 10% SDS-PAGE under reducing (+β-mercaptoehanol) and non-reducing (-β-mercaptoehanol) conditions. Proteins were stained with Coomassie brilliant blue G-250. The human IgG1κ antibody (Sigma Aldrich-Germany) and the IgBCR of MEC-1 human CLL cell line (ACC 497) were included as positive controls. The size marker was 10–250 kD (BioRad Laboratories, Italy). Same procedure was used for the other CLL IgBCRs of patients CLL#1 and CLL5. A representative experiment is shown.
