# Supplementary material for: Detection of chronic lymphocytic leukemia subpopulations in peripheral blood by phage ligands of tumor immunoglobulin B cell receptors
Source: Leukemia. 2020 Jun 1;35(2):610–4. doi: 10.1038/s41375-020-0885-y (PMC7862058; doi:10.1038/s41375-020-0885-y)
Supplement: Supplementary file 5 — Supplementary Figure S3. Workflow of the RPL screening. [file 41375_2020_885_MOESM5_ESM.pdf]

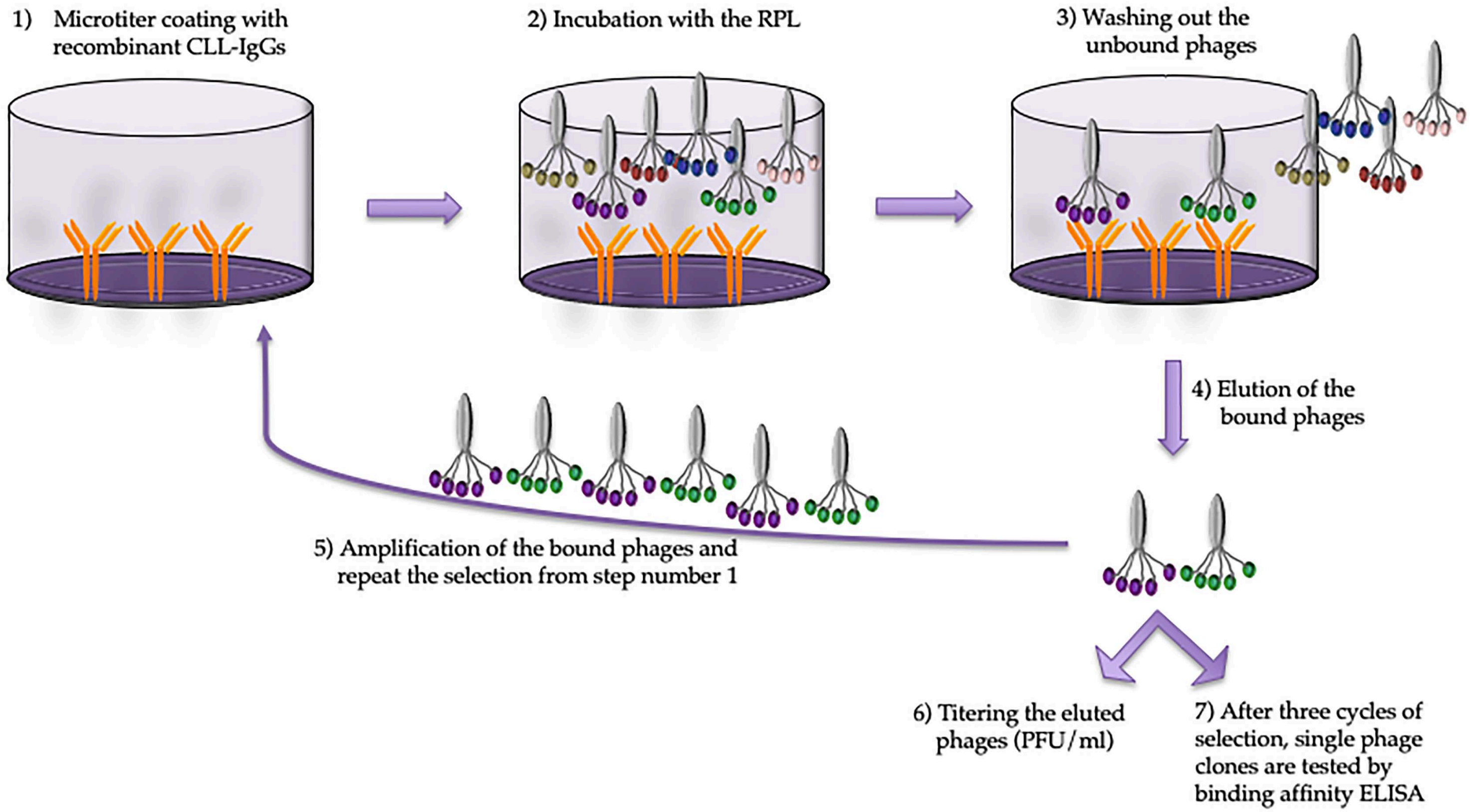

Supplementary Figure S3. Workflow of the RPL screening.

(1) A microtiter was coated with the recombinant IgG corresponding to the VH1-69 CLL-IgBCR in carbonate buffer (pH9.0); (2) The coated plate was 1h- incubated at 37°C with the C7C-Phage Display Library in PBS 1X; (3) Unbound phages were washed out; (4) Bound phages were eluted in elution buffer (pH2.2); (5) Eluted phages were amplified for another cycle of selection; (6) Phages were titered; (7) Phages were analysed by ELISA to determine the affinity binding to the CLL IgBCR.
