# Supplementary material for: Detection of chronic lymphocytic leukemia subpopulations in peripheral blood by phage ligands of tumor immunoglobulin B cell receptors
Source: Leukemia. 2020 Jun 1;35(2):610–4. doi: 10.1038/s41375-020-0885-y (PMC7862058; doi:10.1038/s41375-020-0885-y)
Supplement: Supplementary file 6 — Supplementary Figure S4. Binding assay of phages 1, 2 and 3 to the VH1-69 U-CLL IgG of patient CLL#5. [file 41375_2020_885_MOESM6_ESM.pdf]

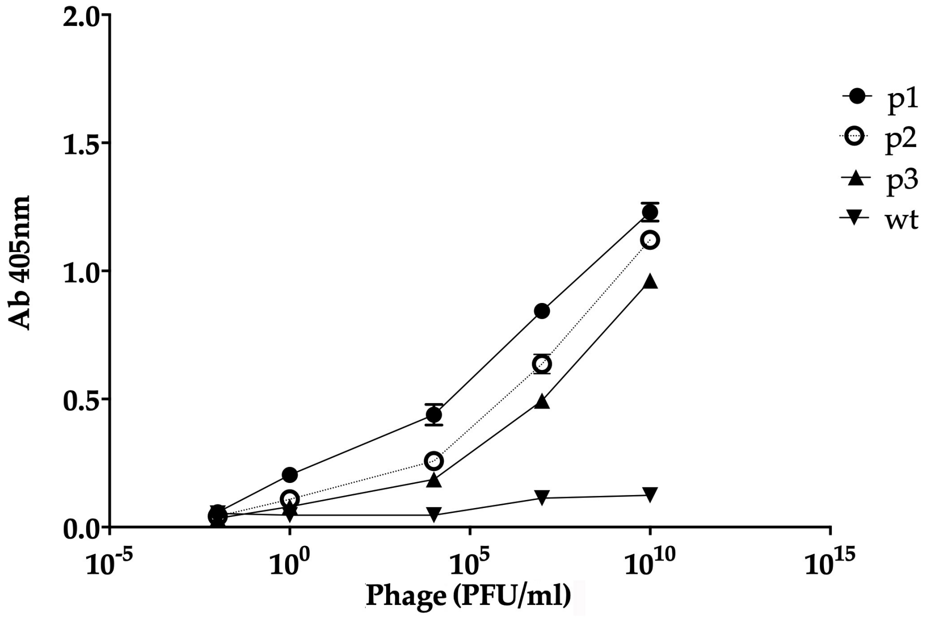

Supplementary Figure S4. Binding assay of phages 1, 2 and 3 to the VH1-69 U-CLL IgG of patient CLL#5.

The purified VH1-69 U-CLL IgG (10ng/µl) of patient CLL#5 was immobilized on 96-well microplate and incubated with the phages p1, p2 or p3 at the indicated concentrations. The wild type (wt) M-13 phage was included as control. Phage binding was analysed by ELISA using the antibody anti-M13 HRP-conjugated and measured by absorbance (Ab) at 405 nm. Absorbance values are reported as the mean ± SEMs of 3 independent experiments.
